# Tyrosine-kinase inhibitors for lung or breast cancer and drug–drug interactions: a clinical guide

**DOI:** 10.3389/or.2025.1612249

**Published:** 2025-09-22

**Authors:** Fiorenza Santamaria, Michela Roberto, Giulia Maltese, Francesco Nicolella, Andrea Torchia, Dorelsa Buccilli, Mattia Alberto Di Civita, Paola Giancontieri, Daniele Marinelli, Alessio Cirillo, Vincenzo Bianco, Monica Verrico, Andrea Botticelli, Daniele Santini

**Affiliations:** ^1^ Department of Experimental Medicine, Sapienza University of Rome, Rome, Italy; ^2^ Division of Medical Oncology, Department of Hematology, Oncology and Dermatology, AOU Policlinico Umberto I, Rome, Italy; ^3^ Department of Radiological, Oncological and Anatomopathological Sciences, Sapienza University, Rome, Italy; ^4^ Clinical and Molecular Medicine, Sapienza - Università di Roma, Rome, Italy; ^5^ Department of Medico-Surgical Sciences and Biotechnologies, Sapienza University of Rome, Rome, Italy

**Keywords:** tyrosine-kinase inhibitors, lung cancer, NSCLC-lung adenocarcinoma—EGFR–ALK–BRAF–KRAS–RET–MET–PD-L1–ROS1, breast cancer, drug–drug interactions, HER2 breast cancer +

## Abstract

**Introduction:**

In the last two decades, tyrosine-kinase inhibitors (TKIs) have dramatically changed the prognosis of lung and breast cancers, with significant benefits in the metastatic setting and, more recently, in the adjuvant setting for selected groups of patients. Despite their favorable oncological effects, TKIs carry a high risk for drug–drug interactions (DDIs) due to their pharmacokinetics (PK), which depend on both pH-dependent absorption and liver metabolism. However, DDIs are frequently related to “potential DDIs” (pDDIs), and their clinical relevance is often underestimated; there is also a lack of practical guidance for clinicians.

**Methods and materials:**

We conducted a narrative review restricted to the last 20 years, involving adult individuals (aged 18 years or older) with lung or breast cancers treated with TKIs and clinical data on potential DDIs, along with reported toxicities or outcomes.

**Results:**

We summarized the pharmacokinetic profiles and the clinical evidence of 11 TKIs used for lung or breast cancers. Moreover, we provided an easy-to-use guide to help physicians in clinical practice with recommended dose adjustments or cautions necessary to prevent severe adverse events or possible changes in TKI availability in the presence of other interfering drugs.

**Conclusion:**

The level of evidence for DDIs during TKI treatment is low because most available data are from phase I studies in healthy volunteers and few phase II studies in cancer patients. However, since the occurrence of DDIs can be clinically significant and a prompt drug reconciliation process can be useful to prevent them, further prospective, large-sample-size clinical trials should be carried out.

## 1 Introduction

Breast cancer is the most frequently diagnosed malignancy worldwide, while lung cancer is the second most commonly diagnosed cancer and the leading cause of cancer-related death globally, accounting for nearly 1.80 million deaths (1). Over the past two decades, tyrosine-kinase inhibitors (TKIs) have revolutionized the treatment paradigm for molecularly selected non-small-cell lung cancer (NSCLC) and HER2-positive breast cancer patients. These molecules improved survival in the metastatic setting and, more recently, have also demonstrated a significant benefit in the adjuvant setting (2–5).

Tyrosine kinases (TKs) are enzymes that regulate key cellular processes, including proliferation, apoptosis, differentiation, and survival, through various signaling pathways. TKs can be transmembrane receptors or cytoplasmic non-receptor proteins. Mutations or overexpression of these proteins may lead to uncontrolled cell proliferation and promote carcinogenesis (6, 7). Thus, TKIs are molecular-targeted therapies that can arrest the uncontrolled signaling pathways responsible for the abnormal proliferation of cancer cells. All TKIs are oral agents with flexible and convenient administration. However, the pharmacokinetics (PK) of TKIs and their bioavailability depend primarily on both absorption and first-pass hepatic metabolism. It is demonstrated that gastrointestinal absorption represents a crucial passage in the pharmacokinetics of TKIs, and factors changing stomach pH or downregulation of cellular transporters [such as P-glycoprotein (P-gp) inhibitors] could significantly affect TKIs’ plasma levels (8). This effect is particularly relevant for TKI molecules with a bioavailability of less than 50% or for those whose activity is influenced by influx or efflux cellular transporters. Moreover, TKIs undergo extensive first-pass hepatic metabolism, primarily mediated by cytochrome P450 (CYP450) enzymes. This characteristic may lead, during the co-administration of TKIs with other drugs, to enhanced or reduced CYP450 activity, potentially resulting in altered serum concentrations of the TKI (8). In clinical practice, the co-administration of drugs that can interfere with the CYP-mediated metabolism of TKIs is very common and should be avoided because of the significant risk of severe adverse events or loss of therapeutic effects (9). However, drug–drug interactions (DDIs) are frequently referred to as “potential DDIs,” and their real clinical impact on oncological outcomes (toxicity and survival) is lacking. We previously reported that polypharmacy may negatively influence toxicities and oncological outcomes in patients treated with selective inhibitors of the cyclin-dependent kinases CDK4 and CDK6 (CDK4–6I), antibody–drug conjugates (ADCs), poly ADP-ribose polymerase inhibitors (PARPis), androgen-receptor targeted agents (ARTAs), and immune-checkpoint inhibitors (ICIs) (10). The purpose of this review is to systematically collect all clinical evidence on DDIs occurring between TKIs used for lung and breast cancers and concomitant medications, with a focus on their impact on oncological outcomes and adverse drug reactions (ADRs), and provide an easy-to-use guide with recommended dose adjustments or necessary precautions.

## 2 Results

Eleven molecules belonging to the TKI class, largely used for lung and breast cancers, are discussed in the present review. The PK profile, therapeutic indications, and a summary of the clinical evidence on DDIs between TKIs and other drugs are summarized in Supplementary Table S1. Starting from these literature data, we provide an easy-to-use guide on the clinical management of DDIs to help physicians avoid unfavorable drug associations that could lead to a loss of efficacy or increased toxicity (Table 1).

**TABLE 1:** Easy-to-use guide about potential DDIs with concomitant medications.

Drug name	It is to be avoided or taken with caution	It is recommended	
Afatinib	Caution is required with strong P-gp inducers	If co-administration with a strong P-gp inducer is unavoidable, the afatinib dose should be increased by 10 mg daily	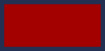
	Caution is required with strong P-gp inhibitors	Co-administration with strong P-gp inhibitors should be 12 or 6 h apart from afatinib, depending on whether P-gp is administered once or twice daily, respectively	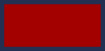
	Co-administration with high-fat meal should be avoided	Food should not be consumed for at least 3 h before and at least 1 h after taking afatinib	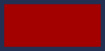
Alectinib	Administration during high-fat meal should be avoided	Alectinib should be administered under fed conditions	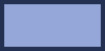
	Caution is advised with P-gp and BCRP substrates	If co-administration with P-gp and BCRP substrates is unavoidable, patients should be monitored for signs of toxicity, especially for drugs with a narrow therapeutic window	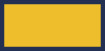
Brigatinib	Co-administration with strong inducers or inhibitors of CYP3A4 should be avoided	If the combination with the CYP3A4 inhibitor is unavoidable, the starting dose of brigatinib should be reduced by 50%	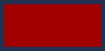
Capmatinib	Co-administration of capmatinib with strong CYP3A inducers should be avoided	If co-administration with strong CYP3A inhibitors is unavoidable, patients should be closely monitored for signs of toxicity	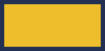
Crizotinib	Co-administration of crizotinib with strong CYP3A4 inhibitors or inducers should be avoided	If co-administration with CYP3A4 inductors is unavoidable, the crizotinib dose should be gradually increased	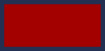
	Caution is recommended in case of co-administration of crizotinib with moderate CYP3A inhibitors or with P-gp substrates with a narrow therapeutic window		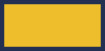
	High-fat food should be avoided		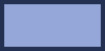
Lapatinib	Concomitant treatment with substances that increase gastric pH should be avoided	Lapatinib should be taken either at least 1 hour before or at least 1 hour after food	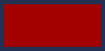
	Co-administration of lapatinib with inducers of CYP3A4, strong inhibitors of CYP3A4, or medicinal products with narrow therapeutic windows that are substrates of CYP3A4 and/or CYP2C8 should be avoided		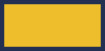
Lorlatinib	Co-administration with strong or moderate CYP3A4/P-gp inducers should be avoided	If co-administration with a strong CYP3A4 inhibitor is necessary, the larotrectinib dose should be reduced by 50%	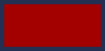
	Co-administration with strong inducers or inhibitors of CYP3A4/5 and CYP3A4/5 substrates with a narrow therapeutic index should be avoided	If co-administration with CYP3A4 inhibitor is unavoidable, the starting dose of lorlatinib should be reduced from 100 mg to 75 mg	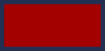
	Caution should be taken with concomitant use of P-gp substrates with a narrow therapeutic index	If co-administration with P-gp substrates is unavoidable, patients should be monitored for signs of toxicity	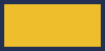
Nintedanib		If co-administration with P-gp potent inhibitors is necessary, patients should be closely monitored for adverse drug reactions	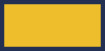
		Nintedanib should be taken with food or during or immediately before or after a meal	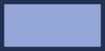
Osimertinib	Caution is recommended in patients with a history of risk factors for QTc prolongation and in those receiving concomitant medicinal products that might prolong the QTc interval	Cardiological monitoring with ECG is recommended 24–48 h before and 1 week after	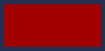
	Co-administration with strong or moderate CYP3A inducers should be avoided		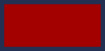
	Caution is recommended when using osimertinib with sensitive BCRP substrates		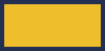
Selpercatinib	Concomitant use of strong CYP3A4 inducers or inhibitors should be avoided	If co-administration with a strong CYP3A4 inhibitor is unavoidable, the selpercatinib dose should be reduced by 50%	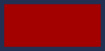 *
	Co-administration with sensitive CYP2C8 and CYP3A4 substrates should be avoided		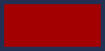
	Caution should be taken when taking a sensitive P-gp substrate, particularly those with a narrow therapeutic index		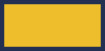
Tucatinib	Concomitant use with strong CYP2C8 and CYP3A4 inhibitors or moderate CYP2C8 inducers should be avoided	If co-administration with a strong CYP2C8 inhibitor is unavoidable, the starting tucatinib dose should be reduced to 100 mg orally twice daily	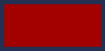
	Co-administration of tucatinib with CYP3A substrates should be avoided	If concomitant use is unavoidable, the CYP3A substrate dosage should be reduced in accordance with the concomitant medicinal product SmPC.	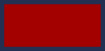

Legend: 
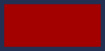
: Concomitant use should be avoided; in case it is unavoidable, the drug dosage should be reduced/increased, the administration should be delayed, or other drugs should be used,

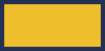
: In case of concomitant use, caution and monitoring for adverse events are advised.

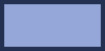
: Warning for the administration of TKIs with/without food.

CYP, cytochrome P450; UGT, uridine 5′-diphospho-glucuronosyltransferase; P-gp, P-glycoprotein 1; BCRP, breast cancer resistance protein.

### 2.1 Lung cancer

#### 2.1.1 Afatinib

Afatinib is a second-generation, irreversible anti-EGFR blocker. Afatinib exhibits high solubility throughout the physiological pH spectrum (1–7.5). As reported by Peters et al., its absorption is not significantly affected by acid-reducing agents, such as antacids, proton-pump inhibitors (PPIs), or anti-H2 inhibitors (11). Furthermore, unlike other EGFR TKIs, which undergo metabolism via cytochrome P450 enzymes, CYP enzymes have minimal involvement in the metabolism of afatinib, so the risk of interactions between afatinib and co-administered medications that undergo CYP enzyme metabolism is minimal (11). *In vitro* studies indicate that afatinib is a substrate and inhibitor of P-gp, implying that concomitant use of strong P-gp inhibitors or P-gp inductors can increase or decrease exposure to afatinib (12). Co-administration of ritonavir, a potent inhibitor of P-gp and BCRP (another ATP-binding cassette drug transporter), did not result in clinically meaningful changes in afatinib exposure in healthy male adults. Conversely, rifampicin, a strong P-gp inducer, significantly decreased afatinib exposure (13). Consequently, when afatinib is co-administered with P-gp inducers, the dose of afatinib should be increased by 10 mg daily. On the contrary, co-administration with P-gp inhibitors should be spaced 12 or 6 h apart from afatinib, depending on whether P-gp inhibitors administered once or twice daily, respectively. To date, no clinical evidence has demonstrated a clinically relevant DDI between afatinib and P-gp substrate (13).

#### 2.1.2 Alectinib

Alectinib is an oral selective anti-anaplastic lymphoma kinase (ALK) inhibitor. An open-label study in healthy subjects (14, 15) demonstrated that the co-administration of omeprazole or esomeprazole with alectinib did not significantly alter the combined exposure of alectinib and M4, indicating that no restrictions are needed when alectinib is used with anti-secretory agents (14). Alectinib undergoes primary hepatic metabolism via cytochrome P450 3A (CYP3A), which converts it into a major, similarly active metabolite, M4, which is subsequently metabolized by CYP3A. It is interesting to note that alectinib and M4 showed weak time-dependent inhibition and minimal induction of CYP3A *in vitro* (16), but according to Morcos et al. (17), co-administration with posaconazole (potent CYP3A4 inhibitor), rifampicin (CYP3A4 inducer), and midazolam (sensitive substrate of CYP3A) did not lead to relevant modifications in alectinib’s AUC or C_max_. Consequently, the weak *in vitro* time-dependent inhibition and induction effects of CYP3A by alectinib and M4 do not translate into any clinically meaningful effects *in vivo*. This finding suggests that alectinib can be administered with a CYP3A inhibitor or inducer without the need for dose adjustments. Since alectinib is a P-gp and BCRP inhibitor, careful monitoring of side effects of these substrates is advised, especially for drugs with a narrow therapeutic window, such as digoxin (18).

#### 2.1.3 Brigatinib

Brigatinib is a second-generation anaplastic lymphoma kinase (ALK) inhibitor. A preclinical *in vitro* study (19) showed that brigatinib is predominantly metabolized by CYP3A4 and CYP2C8, with only minor involvement of CYP3A5. N-demethylation and cysteine conjugation are the two primary metabolic clearance pathways described. Approximately 65% and 25% of brigatinib’s systemic concentrations are eliminated via feces and urine, respectively (20). An *in vitro* pre-clinical study showed that co-administration with gemfibrozil, a strong CYP2C8 inhibitor, resulted in a reduction in brigatinib’s C_max_ and AUC of 41% and 15%, respectively. Hence, no brigatinib dose modifications are required during co-administration with CYP2C8 inhibitors, and no clinically relevant DDI has occurred. Itraconazole significantly increased brigatinib exposure (AUC + 82%), while rifampicin led to an important decrease in brigatinib’s plasma levels (AUC − 80%). These findings suggest a significant impact on brigatinib systemic exposure. This may result in decreased brigatinib efficacy, even though further clinical investigations are needed. Hence, co-administration of strong CYP3A inhibitors and inducers should be avoided, but if unavoidable, a 50% dose reduction in brigatinib is recommended; no dose modification is required with concomitant use of strong CYP3A4 inducers, although dose titration and monitoring for AEs are advised with moderate CYP3A4 inducers (19).

#### 2.1.4 Capmatinib

Capmatinib is an oral, small-molecule TKI targeting the MET (MNNG-experimentally transformed) proto-oncogene, which encodes the receptor for the hepatocyte growth factor (HGF), a key driver of epithelial–mesenchymal transition (EMT). Capmatinib undergoes biotransformation mainly through CYP3A4 and aldehyde oxidase, with excretion primarily in healthy subjects via feces (42%) and secondarily via urine (22%) (21). Similarly to other TKIs, preclinical evidence showed that when capmatinib is co-administered with a strong CYP3A inhibitor, its systemic exposure increases, which could enhance the incidence and severity of AEs. On the other hand, co-administration with a strong or moderate CYP3A inducer decreases capmatinib exposure, which may reduce antitumor activity (22). No clinical evidence suggests capmatinib dose modification with CYP3A4 inhibitors or inducers. A preclinical study showed that capmatinib acts as a reversible inhibitor of cytochrome CYP3A4 and exhibits moderate inhibition of CYP1A2. These effects were associated, in a time-dependent manner, with an increase in midazolam (a substrate of CYP3A4) C_max_ of 22% and caffeine (a CYP1A2 substrate) AUC of 134%. Even though no change in caffeine C_max_ and no increase in AEs were demonstrated after co-administration and the increase in midazolam C_max_ was not clinically relevant (23), no relevant DDIs have been demonstrated so far, and further clinical investigations are needed.

#### 2.1.5 Crizotinib

Crizotinib is a selective small-molecule inhibitor of the ALK receptor tyrosine kinase (RTK) and its oncogenic variants (i.e., ALK fusion events and selected ALK mutations), an inhibitor of the hepatocyte growth factor receptor (HGFR, c-Met) RTK, ROS1 (c-ROS), and receptor d’Origine Nantais (RON) RTK (24). Crizotinib is mainly metabolized by cytochrome (CYP 3A), which also mediates the formation of its main circulating metabolite, lactam PF-06260182 (25). These findings suggest that modulation of CYP3A activity, either through inhibition or induction, may significantly increase or decrease the systemic exposure of crizotinib, respectively, thereby increasing the risk of toxicity or sub-therapeutic exposure. Xiu et al. (26), in a phase 1 trial in healthy volunteers, demonstrated that concomitant administration with a strong CYP3A inhibitor led to 5.2- and 1.6-fold increases in AUC and C_max_, respectively, while a CYP3A inducer caused a marked reduction in crizotinib systemic exposure (AUC by 94% and C_max_ by 89%). Hence, the concomitant administration of crizotinib with strong CYP3A4 inhibitors should be avoided unless the potential benefit to the patient outweighs the risk, in which case the patient should be closely monitored for crizotinib-related adverse events (24). The concomitant administration of dexamethasone (the most commonly used CYP3A inducer) with crizotinib was explored in a *post hoc* analysis from all PROFILE studies (1001, 1005, 1007, and 1014) (27), including 1,690 patients. The study revealed that crizotinib plasma exposure remained similar, regardless of co-administration with dexamethasone, indicating no pharmacokinetic interaction between dexamethasone or other CYP3A inducers with similar potency and crizotinib (27). Preclinical *in vitro* evidence showed that crizotinib strongly inhibits P-gp (28), and therefore, caution must be exercised when administering crizotinib with medicinal products that are substrates of P-gp, especially if they have a narrow therapeutic window, due to increased therapeutic effect or adverse reactions (8, 24). Crizotinib also inhibits OCT1 and OCT2, which may result in increased plasma concentrations of co-administered substrates of OCT1 or OCT2 (e.g., metformin and procainamide) (24). A phase 1 study in healthy volunteers (29) evaluated the effect of multiple doses of the PPI esomeprazole on the pharmacokinetics and safety of crizotinib. It showed that, in the PPI arm, there was an approximately 10% decrease in crizotinib total exposure (AUC_inf_) and no change in peak exposure (C_max_) (29). Therefore, although esomeprazole decreased total exposure of crizotinib, it is not considered clinically meaningful, and dose modification is not required when crizotinib is co-administered with agents that affect gastric pH (such as proton-pump inhibitors, H2 blockers, or antacids) (24). Finally, crizotinib may reduce the heart rate, an effect that could develop several weeks after the start of treatment. In this case, caution is warranted when crizotinib is used alongside other bradycardic agents (e.g., beta-blockers, non-dihydropyridine calcium channel blockers, clonidine, and digoxin) (24).

#### 2.1.6 Lorlatinib

Lorlatinib is an oral small-molecule inhibitor of ALK and ROS1 kinase. A food-effect study in healthy volunteers showed that administration of rabeprazole resulted in a 29% increase in lorlatinib C_max_, although no other relevant DDIs have been identified with other acid-suppressive agents (30). The major pathways for lorlatinib metabolism are phase I oxidation and glucuronide conjugation, which produce PF-06895751, the main pharmacologically inactive metabolite. *In vitro* preclinical studies have shown that lorlatinib is predominantly metabolized by CYP3A and UGT1A4, while CYP2C19, CYP2C8, and UGT1A3 play only minor roles (31). According to a clinical study conducted in healthy volunteers, co-administration with rifampicin, a strong CYP3A4/5 inducer, led to a decrease in lorlatinib AUC and C_max_ by 85% and 76%, respectively, with an increased risk of hepatotoxicity (elevated level of AST/ALT with no modification of other hepatic indices of any grade according to CTCAE) within 3 days from administration. Consequently, co-administration with strong CYP3A4/5 inducers (rifampicin, carbamazepine, enzalutamide, mitotane, phenytoin, and St. John’s Wort) may significantly reduce plasma concentrations of lorlatinib and should be avoided (32). Another clinical study in healthy volunteers suggests that lorlatinib could be safely co-administered with moderate CYP3A inducers such as modafinil, without notable changes in AUC, C_max_, and AEs (33). Furthermore, Patel et al. demonstrated in healthy volunteers that the strong CYP3A4 inhibitor itraconazole elevated lorlatinib AUC and C_max_ by 141.79% (90% CI, 128.71%, and 156.21%) and 124.39% (90% CI, 110.20%, and 140.41%), respectively, although this was not associated with an increased risk of AEs. Hence, lorlatinib co-administration with strong CYP3A4 inhibitors may lead to a relevant DDI and should be avoided; therefore, if this combination is unavoidable, the starting dose of lorlatinib should be reduced from 100 mg to 75 mg (34). Furthermore, *in vitro* evidence suggests that lorlatinib has moderate CYP3A4/5 inductor activity, with a reduction of 50% in midazolam (a CYP3A4 substrate) C_max_ and a 61% reduction in AUC. Therefore, co-administration of lorlatinib with CYP3A4/5 substrates that have a narrow therapeutic index should be avoided because their concentrations may be reduced by lorlatinib (35). Furthermore, *in vitro* investigations demonstrated that lorlatinib exerts weak inhibitor activity on CYP2B6, UGT1A1, and CYP2C19 and moderate P-gp induction activity. These properties did not demonstrate clinically relevant DDIs, and consequently, no lorlatinib dose modifications are required when it is co-administered with drugs metabolized by CYP2B6, UGT1A1, and CYP2C19. Concomitant use of P-gp substrates with a narrow therapeutic index (digoxin or dabigatran) should be approached with caution when combined with lorlatinib, and careful monitoring of AEs is required (36).

#### 2.1.7 Nintedanib

Nintedanib is a small-molecule tyrosine kinase inhibitor targeting the platelet-derived growth factor receptors (PDGFRs) α and β, the fibroblast growth factor receptor (FGFR) 1–3, and VEGFR1–3 (37). Nintedanib metabolism and excretion are mainly mediated by liver UGT1A1, while CYP3A4 enzymes exhibit only a minor involvement. Therefore, interactions between nintedanib and other drugs modulating CYP enzymes are expected to be minimal. Nintedanib is a substrate of P-gp, and according to a phase I study in healthy subjects (38), co-administration of ketoconazole (a strong P-gp inhibitor) led to an increase in nintedanib C_max_ and AUC by 160.5% and 160.5%, respectively; conversely, rifampicin (a strong P-gp inducer) decreased its C_max_ and AUC by 59.8% and 50.1%, respectively. As a result, careful monitoring for side effects is advised when nintedanib is administered with a strong P-gp inhibitor, although dose adjustment is not typically required (37). On the other hand, given that potent P-gp inducers (e.g., rifampicin, carbamazepine, phenytoin, and St. John’s Wort) significantly lower nintedanib exposure, their concurrent use should be carefully considered (38). No other evidence on potential nintedanib DDI is available, and further investigations are needed.

#### 2.1.8 Osimertinib

Osimertinib is a third-generation EGFR inhibitor that binds irreversibly to EGFRs harboring sensitizing mutations (EGFRm) and the T790M TKI-resistance mutation (39). Osimertinib is both a substrate and an inhibitor of P-gp and BCRP. In a phase I study, Harvey et al. (40) showed that, when administered with osimertinib, rosuvastatin (a sensitive BCRP substrate) and simvastatin (substrate of P-gp), C_max_ showed significant increases of 72% and 23%, respectively. Therefore, careful monitoring is recommended when osimertinib is combined with sensitive BCRP substrates that have a narrow therapeutic index, and patients should be closely monitored for signs of changed tolerability (39). Furthermore, since CYP3A4 and CYP3A5 are primarily responsible for osimertinib metabolism, its pharmacokinetics could be modified when co-administrated with strong CYP3A4 and CYP3A5 inducers or inhibitors. A phase 1 trial demonstrated that the co-administration of osimertinib with strong CYP3A4 inducers resulted in a significant change in both AUC and C_max_ in contrast to itraconazole (a strong CYP3A4 inhibitor) (41). Therefore, CYP3A4 inhibitors appear to have a minimal impact on osimertinib exposure, while concomitant use of strong CYP3A inducers (e.g., phenytoin, rifampicin, and carbamazepine) with osimertinib should be avoided (39). Occhipinti M. et al. (42) conducted a retrospective analysis to assess the prevalence of DDI in patients undergoing EGFR-TKI. The most frequent co-administration of osimertinib was with SSRIs, antipsychotic drugs, or calcium antagonists, and that association may lead to cardiac rhythm alterations and increase the risk of QTc prolongation (42). Therefore, in patients with congestive heart failure, electrolyte abnormalities, or those who are taking drugs known to prolong the QTc interval, periodic monitoring with electrocardiograms (ECGs) and electrolytes is advisable during osimertinib therapy (39). A phase I study in NSCLC patients (14) assessed the impact of food or PPI use (omeprazole) on the exposure and safety/tolerability of osimertinib, and it demonstrated that the administration of omeprazole or food did not affect osimertinib exposure. Therefore, gastric pH-modifying agents can be concomitantly used with osimertinib without any restrictions (39).

#### 2.1.9 Selpercatinib

Selpercatinib is a RET receptor tyrosine kinase inhibitor (43). Due to its higher selectivity for RET over other tyrosine kinases, selpercatinib is believed to have a more favorable safety profile than other multi-kinase inhibitors; however, the most frequent adverse events associated with selpercatinib treatment are teratogenicity, liver toxicity, hypertension, QTc prolongation, bleeding, and delayed wound repair (44). Selpercatinib undergoes hepatic metabolism mediated by the cytochrome P450 system, primarily CYP3A4, and is, therefore, vulnerable to drug–drug interactions with medications that either inhibit or stimulate the reactivity of the CYP3A4 enzyme (43). In a phase I study in healthy volunteers, repaglinide, a CYP2C8 substrate, showed a 91% increase in C_max_ and a 188% increase in AUC following co-administration with selpercatinib; therefore, it is best to avoid simultaneous administration with sensitive CYP2C8 substrates such as enzalutamide and paclitaxel (45).

### 2.2 Breast cancer

#### 2.2.1 Lapatinib

Lapatinib is a tyrosine-kinase inhibitor that targets human epidermal growth factor receptor type 2 (HER2/ERBB2) and epidermal growth factor receptors (HER1/EGFR/ERBB1) (46). Lapatinib undergoes extensive hepatic metabolism mainly through CYP3A4 and CYP3A5 enzymes, with minor contributions from CYP2C19 and CYP2C8. When lapatinib and irinotecan are administered together, the AUC of SN-38, the active metabolite of irinotecan, increases by approximately 40%; however, the specific mechanism of this interaction remains unclear (47). A study on 17 mBC HER2+ patients showed that esomeprazole treatment resulted in decreased lapatinib bioavailability by 6%–49% (48). Koch et al., in an open-label, nonrandomized, crossover study, showed that lapatinib led to a 2-fold increase in digoxin C_max_ and a 60%–80% increase in AUC; therefore, co-administration of lapatinib with drugs known to have a narrow therapeutic index and that are substrates of ABCB1 should be undertaken with caution, possibly considering dose adjustment (49). Because of its inhibitory effect on CYP2C8 and/or P-gp, simultaneous administration of lapatinib with intravenous paclitaxel enhanced the paclitaxel exposure by 23% (46). A phase III, randomized, double-blind trial reported that the combination of lapatinib with paclitaxel increases the frequency and severity of neutropenia and diarrhea (50).

#### 2.2.2 Tucatinib

Tucatinib decreases the phosphorylation of both HER-2 and HER-3, thereby disrupting MAPK and AKT signaling pathways and inhibiting cell proliferation (51). Tucatinib is primarily metabolized by CYP2C8 and CYP3A. To assess potential DDIs between tucatinib and molecules that are CYP2C9 substrates, a phase I multicenter open-label trial in healthy subjects (52) showed that tucatinib had no effect on the pharmacokinetics of drugs with a CYP2C9-mediated metabolism, and it was well tolerated when administered alone or in combination with other medications. Moreover, tucatinib is a strong inhibitor of CYP3A, a weak inhibitor of CYP2C8 and P-gp, and an inhibitor of UGT1A1 (52). When co-administration of tucatinib with strong CYP2C8 inhibitors (e.g., gemfibrozil, clopidogrel, sorafenib, erlotinib, and dabrafenib) is unavoidable, a dose reduction from 150 mg to 100 mg twice daily is recommended (51).

## 3 Discussion and conclusion

This review analyzed the pharmacokinetic profile and the available clinical evidence of the occurrence of DDIs that have been described in the literature with 11 types of TKIs used in lung and breast cancers. TKIs are orally administered drugs whose solubility—and consequently plasma exposure—is influenced by gastric pH and metabolism via CYP450 enzymes. Chronic use of PPIs can reduce gastric acidity, potentially impairing the absorption—and, therefore, therapeutic efficacy—of pH-sensitive TKIs, such as afatinib, alectinib, and lapatinib (53). Given the frequent overuse of PPIs for symptomatic treatment of epigastric pain or heartburn, it is fundamental to evaluate the concomitant use of PPIs in cancer patients before starting TKI to avoid any alternations in absorption. The current relevance of DDIs in patients treated with TKIs has also been extensively described in hematology (54), and for example, in patients with chronic myeloid leukemia (CML), potential DDIs can greatly impact therapy due to significant interpatient variability in the PK of TKIs (54, 55). Despite all these studies, it turned out that the level of evidence for DDIs during TKI treatment is low because the majority of available data are from phase I registration trials (often conducted in healthy volunteers) and few phase II trials enrolling cancer patients. The main studies, including large cohorts of patients, derive from a *post hoc* analysis from all PROFILE studies evaluating crizotinib and dexamethasone co-administration (27), a randomized phase III trial analyzing the simultaneous administration of lapatinib with paclitaxel (50), and a retrospective real-word study assessing the effects of co-administration of osimertinib with SSRIs, quetiapine, or calcium channel blockers (42). To date, we have identified only two ongoing trials in patients with CML assessing the pharmaceutical conciliation of concomitant treatments with TKIs (NCT05130138 and NCT05259228); no similar studies were found in solid tumor settings. Real-world data (RWD) and real-world evidence (RWE) have become increasingly important in post-marketing pharmacovigilance and informing confirmatory trials to address clinically relevant questions (56). Ferrer et al., in a recent review on the effects of adherence, food intake, and pharmaceutical form on the PK of TKIs used in CML, NSCLC, and melanoma, highlighted the importance of therapeutic drug monitoring (TDM) in reducing PK-associated variability (57).

In conclusion, this review provides specific recommendations to guide physicians through the process of managing DDIs during treatment with TKIs in daily clinical practice (Table 1), underscores the unmet need for the proper management of concomitant medications in cancer patients treated with TKIs, and provides data to design additional research focused on the evaluation of the clinical impact of a comprehensive assessment of concomitant medications, herbal supplements, lifestyle factors, and SNPs on oncological treatment outcomes. Further investigations and multicenter RWD are needed to help physicians in prompt knowledge of potential DDI risks and better understand the role of the medication reconciliation process.

## 4 Materials and methods

We conducted a narrative review to provide a summary of all the clinical evidence of DDI involving TKI treatment and co-medication in cancer patients. Databases (PubMed and Google Scholar) were searched in October and November of 2024 for a literature review. The following key terms were used: drug–drug interaction(s), DDI, polypharmacy, medication reconciliation, and concomitant medication (or) administration. The above terms were searched in combination with the following terms: TKI, tyrosine-kinase inhibitors, and the single medical product (i.e., afatinib and alectinib, among others). The research was restricted to studies published in the last 20 years, involving adults (aged 18 years or older), written in English, and conducted in human populations with solid tumors that included an analysis of outcomes in the presence of DDIs. Included articles were phase 1,2, and 3 studies; systematic reviews; meta-analyses; or *post hoc* analyses of randomized controlled trials, nonrandomized controlled trials, and clinical retrospective or prospective clinical studies. Case reports, abstracts, *in vitro* studies, and descriptive studies that did not report nor correlate the presence of DDIs with clinical outcomes were excluded. After reading the articles in full and verifying the main topic of the research, the articles that did not meet the inclusion criteria were excluded. The references of the selected articles were analyzed to identify other articles that met the previously established criteria and that were not located in the consulted databases. Other articles and data sources (e.g., prescribing information) not identified through the PubMed and Google Scholar searches were added, as considered appropriate by the authors, to ensure a comprehensive narrative review.
