# Supplementary material for: Tyrosine-kinase inhibitors for lung or breast cancer and drug–drug interactions: a clinical guide
Source: Oncol Rev. 2025 Sep 22;19:1612249. doi: 10.3389/or.2025.1612249 (PMC12497703; doi:10.3389/or.2025.1612249)
Supplement: Supplementary file 1 [file Table1.docx]

| **Table S1**  **Pharmacokinetic profile, therapeutic indications of TKIs and clinical evidence about DDIs between TKIs and other drugs** | | | | | |
| --- | --- | --- | --- | --- | --- |
| **Drug name** | **Absorption** | **Distribution** | **Elimination** | **Therapeutic indications** | **Clinical evidence** |
| **Afatinib** | Cmax 2-5  hours  Take at least 1 hour before or 2 hours after a meal  Coadministration with high feat meal should be avoided | Plasma protein  binding  95% | Mainly  via  the faeces,  renal  elimination less  than 5%;  half-life: 37 hours; steady state  after 8 days | As monotherapy, is approved for the treatment of EGFR TKI-naïve adult patients with locally advanced or metastatic NSCLC with activating EGFR mutations | Phase I study (Wind et Al, 2014) ritonavir group (N= 24): No significative Afatinib AUC or Cmax modification; rifampicin group (N=22) decrease in Cmax and AUC of afatinib |
| **Alectinib** | Cmax 4  hours  Avoid high-fat meal and take under fed conditions | Plasma protein  binding  99% | Mainly  via  the faeces,  renal  elimination  less than 1%;  half-life: 33 hours; steady state  after 7 days | It is used as first line of treatment in ALK+, unresectable, advanced or recurrent NSCLC | Phase 2 study (Morcos et al, 2016) (N= 58): No relevant Alectinib and M4 AUC and Cmax modifications with rifampicin and Posaconazole. |
| **Brigatinib** | Cmax 1 - 4  Not  significantly  altered by food | Plasma protein  binding  91% | Mainly  via  the faeces,  renal  elimination  25%;  half-life: 25 hours | It is approved for the treatment of ALK-positive advanced or metastatic NSCLC after progression disease on or intolerance to Crizotinib. | No clinical evidence available |
| **Capmatinib** | Cmax 1 - 2  hours  Not  significantly  altered by food | Plasma protein  binding  96% | Mainly  via  the faeces,  renal  elimination  22 %;  half-life 6.5 hours;  steady state  after 15 days | It is indicated for the treatment of advanced or metastatic NSCLC, progressive disease, after first line platinum-based chemotherapy or immunotherapy, with selective MET dysregulation including the mutant variant produced by exon 14 skipping. | No clinical evidence about relevant DDI is available |
| **Crizotinib** | Cmax 4-6  hours  Not  significantly  altered by food | Plasma protein  binding  91% | Mainly  via  the faeces,  renal  elimination  22%;  half-life: 42 hours;  steady state  after 49 doses | As monotherapy, it is indicated for the first-line treatment of adults with ALK-positive advanced NSCLC, for the treatment of adults with previously treated ALK-positive advanced NSCLC, and for the treatment of adults with ROS1-positive advanced NSCLC. | Phase 1 study [Xiu et al. 2015]: (N=15) concomitant administration of a strong CYP3A inhibitor and strong CYP3A inducer significantly increased (AUC and Cmax increased to 5.2- and 1.6-fold) and decreased (AUC 94% and Cmax 89%) Crizotinib systemic exposure, respectively  Post hoc analysis from all PROFILE studies (1001, 1005, 1007, and 1014) [Lin et al 2019], (N=1690) crizotinib plasma exposure was similar in dexamethasone-group and no-dexamethasone-group.  A phase 1 study [Xu et al 2022] (N=16): esomeprazole slightly decreased crizotinib AUCinf and no change in Cmax. |
| **Lapatinib** | Cmax 4 hours  Bioavailability increased up to about 4 times by food, depending on the fat content in the meal.  Lapatinib absorption may be decreased by concurrent treatment with drugs that raise stomach pH.. | Plasma protein  binding  99% | Mainly  via  the faeces; Half-life 14.2 hours; Steady-state after 6-7 days | It is recommended in addition to capecitabine in patient with advanced or metastatic HER2-positive breast cancer who have previously undergone with anthracyclines, taxanes, and trastuzumab treatments. | Open label, single sequence study (Koch KM et al. 2013): esomeprazole (n=16): decreased Lapatinib AUC and Cmax  Lapatinib (n=17): increased Digoxin AUC and AUC.  Phase III, randomized, double-blind study (Guan Z et al. 2013): simultaneous administration of lapatinib (n=444): with intravenous paclitaxel improved the exposure of paclitaxel. |
| **Lorlatinib** | Cmax 1,2  hours  Not  significantly  altered by food | Plasma protein  binding  66% | Mainly via urine (48%) and 41% via the faeces; half-life: 24 hours;  steady state  after 15 days | It is approved as a third generation ALK-TKI for treatment of ALK-positive advanced or metastatic NSCLC after progression or intolerance to first or second ALK inhibitors as Alectinib, Ceritinib or Crizotinib. Lorlatinb is specifically developed to penetrate the blood–brain barrier and have broad potency against common ALK-resistance mutations. | Phase I study (Chen et Al., 2020 N=12): rifampicin decrease in AUC and Cmax of Lolratinib increasing risk of hepatotoxicity.  Phase I (Patel et Al., N=12): Itraconazole increase in AUC and Cmax of Lorlatinib.  Phase I/II study (Chen et Al., 2021 N= 54 single dose -274 multiple doses ): lorlatinib decrease in Cmax and AUC of Midazolam |
| **Nintedanib** | Cmax 2 - 4  hours  It is recommended to take nintedanib with food. | Plasma protein  binding  97% | Mainly  via  the faeces,  renal  elimination less than 1%;  half-life 10-15 hours;  steady state  after 7 days | It is approved in combination with docetaxel for the treatment of locally advanced, metastatic, or locally recurrent non-small cell lung cancer (NSCLC) with adenocarcinoma histology after first-line chemotherapy., Nintedanib is used for the treatment of idiopathic pulmonary fibrosis, systemic sclerosis-associated interstitial lung disease, and interstitial lung disease with progressive phenotype. | Phase I study (Luedtke et Al., 2018 N=34): Ketoconazole and rifampicin increase in AUC and Cmax of Nintedanib. |
| **Osimertinib** | Cmax 6  hours  Not  significantly  altered by food | Plasma protein  binding  93% | Mainly  via  the faeces,  renal  elimination  14%;  half-life: 48 hours; steady state  after 15 days | It is approved for the adjuvant treatment after complete tumour resection in adult patients with stage IB-IIIA NSCLC whose tumours have epidermal growth factor receptor (EGFR) exon 19 deletions or exon 21 (L858R) substitution mutations, for the first-line treatment of adult patients with locally advanced or metastatic NSCLC with activating EGFR mutations, and for the treatment of adult patients with locally advanced or metastatic EGFR T790M mutation-positive NSCLC. | Phase 1 study (Harvey et Al., 2018) (N=58): Osimertinib increase in rosuvastatin and simvastatin AUC and Cmax.  Phase 1 study (Vishwanathan et al.,) rifampicin group (N=40): significatively reduce Osimertinib AUC and Cmax; itraconazole group (N=36) no significative change in AUC and Cmax of Osimertinib.  Retrospective real word study (Occhipinti et al., 2018) (N=92 patients) Osimertinib coadministration with SSRI, quetiapine or calcium antagonist increase risk of QTc interval prolongation. |
| **Selpercatinib** | Cmax 2 hours  Not influenced by food. | Plasma protein  binding  97% | Mainly via the feces (69%) and urine (24%)  Half-life 22 hours; Steady-state after 7 days | It is approved to treat RET-driven non-small cell lung cancer (NSCLC), medullary thyroid cancer and thyroid cancer. | Phase 1, trial in healthy subjects (NCT05469113 2019): Selpercatinib (n=13) increased Repaglinide Cmax and AUC. |
| **Tucatinib** | Cmax 1 - 3 hours  Not influenced by food. | Plasma protein  binding  97% | Mainly via the feces (86%) and via urine 4%; Half-life 5.4 hours;  Steady-state after 4 days | It is approved in association with trastuzumab and capecitabine in unresectable or metastatic HER-2 positive breast cancer. | Phase 1, Open-Label, Fixed-sequence in healthy subjects (Topletz-Erickson A et al.2022): Itraconazole (n=28) increased Tucatinib AUC and Cmax.  Rifampin (n=28) decreased Tucatinib AUC and Cmax. Gemfibrozil (n=28) increased Tucatinib AUC and Cmax.  Tucatinib in combination with Tolbutamide/Midazolam (n=17) increased Midazolam AUC and Cmax.  Tucatinib in combination with repaglinide (n=15) increased repaglinide AUC and Cmax.  Tucatinib in combination with Digoxin (n=13) increased Digoxin AUC and Cmax. |
| **Legend**:  Cmax: the maximum (or peak) serum concentration; AUC: area under the curve; EGFR: epidermal growth factor receptor; TKI: Tyrosine kinase inhibitors; NSCLC; non-small cell lung cancer; ALK: Anaplastic lymphoma kinase; RCC: renal cell carcinoma; BRAF: V-Raf Murine Sarcoma Viral Oncogene Homolog B; HCC: hepatocellular carcinoma; GIST: gastrointestinal stromal tumours; MET: mesenchymal-epithelial transition factor; ROS1: c-ros oncogene 1; CRC: colorectal cancer; MLNs: myeloid/lymphoid neoplasms; FGFR1: Fibroblast Growth Factor Receptors; VEGFR: Vascular Endothelial Growth Factor Receptor; mTOR: mammalian target of rapamycin; IFN-α: interferon-alpha; HER: Human Epidermal Growth Factor Receptor; IL-2: interleukin-2. | | | | | |
